# Measurement and Analysis of Vibronic Coupling in Two Dysprosium(III) Complexes of Opposite Magnetic Anisotropy

**DOI:** 10.1002/chem.202503558

**Published:** 2025-12-12

**Authors:** Yasmin L. Whyatt, Jack Emerson‐King, George F. S. Whitehead, David P. Mills, Stuart K. Langley, Mykhaylo Ozerov, Nicholas F. Chilton

**Affiliations:** ^1^ Department of Chemistry The University of Manchester Manchester UK; ^2^ Division of Chemistry Manchester Metropolitan University Manchester UK; ^3^ National High Magnetic Field Laboratory Florida State University. Tallahassee Florida USA; ^4^ Research School of Chemistry The Australian National University Canberra Australia

**Keywords:** magnetic anisotropy, magnetospectroscopy, quantum chemistry, vibronic coupling

## Abstract

The loss of magnetic memory in single‐molecule magnets (SMMs) is caused by the coupling of molecular vibrations to spin states, which plays a significant role in magnetic relaxation processes. Gaining direct evidence of vibronic coupling using experimental techniques is critical to understanding and controlling this phenomenon. Most studies focus on assessing the spin‐phonon coupling in SMMs to help control this relaxation; herein we gain insight by comparing the SMM [Dy(OPCy_3_)_2_(H_2_O)_5_][CF_3_SO_3_]_3_.2(OPCy_3_) to the non‐SMM [Dy{N(SiMe_3_)_2_}_3_] through collection of far‐infrared magnetospectroscopy (FIRMS) spectra and validation with *ab initio* calculations. Single‐crystal measurements display a prominent feature in the spectra at 340 cm^−1^, corresponding to an electronic excitation which varies depending on the direction of external magnetic field applied. These findings demonstrate the complicated effect of magnetic anisotropy on the vibronic coupling in SMMs and demonstrate the power of FIRMS to study these effects.

## Graphical abstract

Single‐crystal far‐infrared magnetospectroscopy is used to reveal magnetic anisotropy and vibronic coupling in a pair of dysprosium(III) complexes.

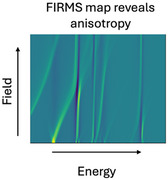

## 1 Introduction

Single‐molecule magnets (SMMs) continue to spark interest in the field of molecular magnetism due to their superparamagnetic behavior, which allows the retention of magnetisation at low enough temperatures in the absence of an external magnetic field, hence an ability to store information at the molecular level [1]. Monometallic lanthanide complexes remain a popular synthetic target owing to the shielded nature of the 4f orbitals, giving rise to free‐ion‐like orbital angular momentum and thus an inherent disposition toward large magnetic anisotropy, with dysprosium(III) complexes in particular dominating the literature [2]. In a seminal paper, Rinehart and Long described how the coordination environment of a lanthanide complex can be designed around the characteristic 4f electron density of the chosen metal ion to achieve a desired magnetic anisotropy [3, 4].

Easy‐axis magnetic anisotropy can be achieved with dysprosium(III) by enforcing a (near‐)linear two‐coordinate geometry [5], a design finally realised experimentally only recently [6, 7], or a sandwich‐type geometry typified by dysprosocenium ions and related families of complexes [7, 8, 9, 10, 11], where the axial crystal field splitting causes the largest $m_{J}$ projections of the lowest‐lying total angular momentum *J* = 15/2 to be stabilized relative to the smallest $m_{J}$ projections. This easy‐axis anisotropy is synonymous for SMMs with a large energy barrier to the reversal of magnetization (*U*_eff_) between one of the degenerate $\pm m_{J}$ magnetic states to the other $\mp m_{J}$. Almost all approaches to enhancing the performance of SMMs revolve around increasing the height of this energy barrier, over which the molecules must traverse via the Orbach process [12, 13], to prolong magnetic memory. However, theoretical studies indicate that increasing the value of *U*_eff_ for monometallic dysprosium(III) SMMs may have reached a plateau [14].

Consequently, if the memory timescale of SMMs is to be enhanced further, other strategies are required. As the magnetization reversal process (for either the sequential single‐phonon Orbach process or the two‐phonon Raman scattering process) [15, 16, 17] is dictated by spin‐phonon (vibronic) coupling, engineering either the phononic degrees of freedom or the spin‐phonon coupling itself is a possible route forwards. Before these interactions can be controlled, we need a comprehensive picture of spin‐phonon coupling in molecules. However, this is a nontrivial task mainly due to the difficulties in observing vibronic states. As such, first principles calculations could prove invaluable as rationalization and design tools, but the sheer number of phononic degrees of freedom in molecular crystals and the correlated nature of the 4f electrons makes these calculations very challenging [15].

Several experimental efforts have been undertaken to obtain direct evidence on spin‐phonon coupling [18, 19], along with contemporary theoretical efforts [20, 21, 22]. All of these experimental reports have utilized far‐infrared magnetospectroscopy (FIRMS), which probes the magnetic‐field dependence of the FIR spectrum [23, 24]. FIRMS is able to directly explore the connection of phonons to electronic states and has thus far been mainly employed to study transition metal compounds [18, 21, 22, 25, 26, 27, 28, 29, 30, 31, 32, 33]. More recently it has been used to study lanthanide compounds [20, 34], including work by some of us in probing the origin of vibronic transitions in a [Yb(trensal)] (H_3_trensal = 2,2,2‐tris(salicylideneimino)triethylamine) molecular qubit [20]. In that work, we also developed a fully *ab initio* theoretical approach to simulate the FIRMS spectrum. The FIRMS experiment can be incredibly data‐rich and provide direct insight into the spin‐phonon coupling that is only inferred in measurements of the magnetic reversal rates of SMMs. In this way, preparation of a high‐quality first‐principles simulation of a detailed FIRMS spectrum is a high‐bar for benchmarking the quality of underlying theoretical methods for calculating spin‐phonon coupling. Subsequently, adequately benchmarked calculations could be used to ask questions that cannot be answered directly in experiment, such as determining which phonon modes dominate magnetic reversal processes in SMMs.

**FIGURE 1:**
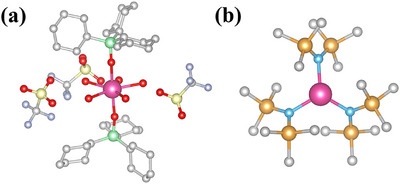
Molecular structures of **1** (a) and **2** (b). Color scheme: Dy, pink; O, red; P, green; S, yellow; F, pale blue; N, bright blue; Si, orange; C, grey. H‐atoms and some counterions are omitted for clarity.

Herein, we study two previously‐reported dysprosium(III) complexes with opposite senses of magnetic anisotropy: [Dy(OPCy_3_)_2_(H_2_O)_5_][CF_3_SO_3_]_3_·2(OPCy_3_) (**1**, OPCy_3_ = tricyclohexylphosphine oxide), a seven‐coordinate pentagonal bipyramidal SMM with easy‐axis anisotropy [35], and [Dy{N(SiMe_3_)_2_}_3_] (**2**), a three‐coordinate trigonal pyramidal compound with easy‐plane magnetic anisotropy (Figure 1) [36]. For **1**, which is air stable, we furthermore perform the single‐crystal FIRMS experiment to probe anisotropy in the vibronic coupling. We find that different external magnetic field directions relative to the single‐crystal of **1** cause a significant variation of features in the FIRMS spectra.

## 2 Results and Discussion

**FIGURE 2:**
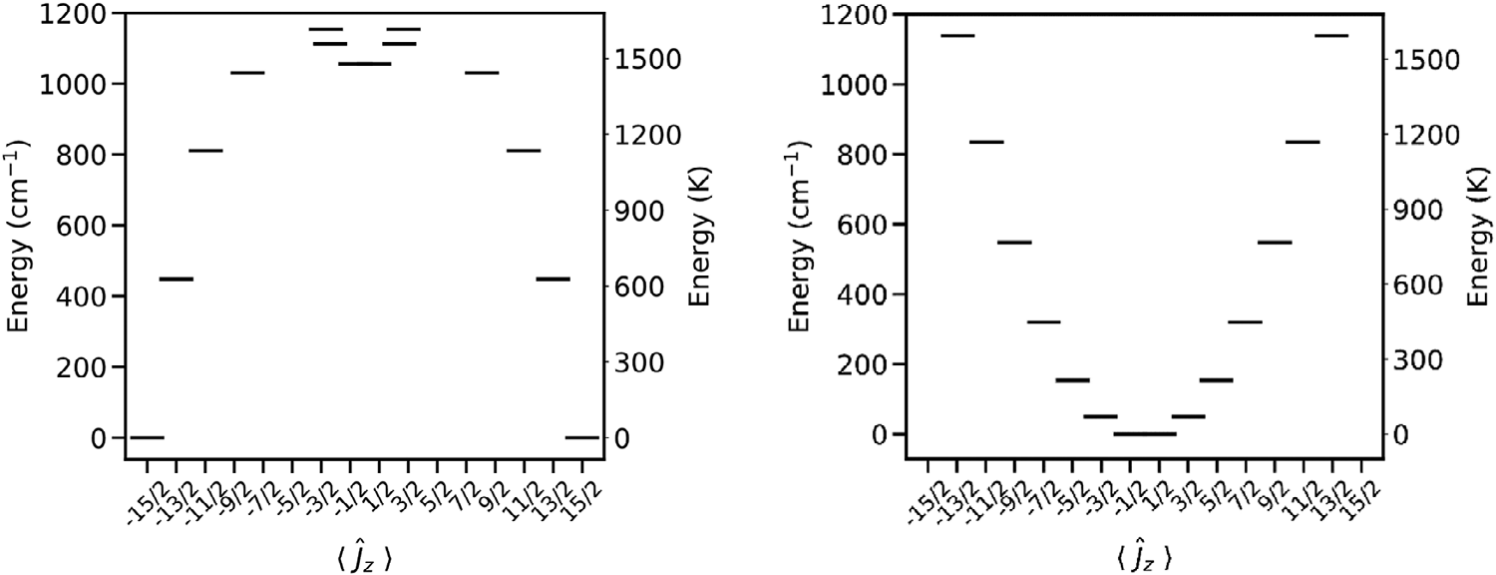
Electronic states of the ground ^6^H_15/2_ multiplet obtained with CASSCF‐SO calculation using the crystal geometries of **1** (left) and **2** (right).

Complex **1** crystallizes in the monoclinic space group *P*2_1_ and exhibits SMM behavior with a modest *U*_eff_ value of 390(5) cm^−1^ [35]. In contrast, complex **2** crystallizes in the high‐symmetry trigonal space group, *P*‐31*c* and does not show any SMM properties [37, 38]. A preliminary calculation of the electronic structures of **1** and **2** with complete active space self‐consistent field spin‐orbit (CASSCF‐SO) methods (gas‐phase molecules using geometries from X‐ray diffraction with the OpenMolcas package [39, 40], see SI for details) shows a crystal field splitting as expected for a SMM for **1**, with a ground doublet well described as $m_{J}=\pm$15/2 (*g*_x_ = *g*_y_ = 0.00 and *g*_z_ = 19.98; $m_{J}$ and *g*_z_ quantised along the Cy_3_PO–Dy–OPCy_3_ axis, Figure S1) and a first excited $m_{J}=\pm$13/2 state at 451 cm^−1^ (Figure 2, Table S1). Near‐opposite crystal field splitting is obtained for complex **2**, possessing a $m_{J}=\pm$1/2 ground state (quantised along the C_3_ symmetry axis) and a substantially smaller energy gap to the first excited state of 51 cm^−1^ (Figure 2, Table S2); both results are consistent with previous findings [35, 41, 42].

**FIGURE 3:**
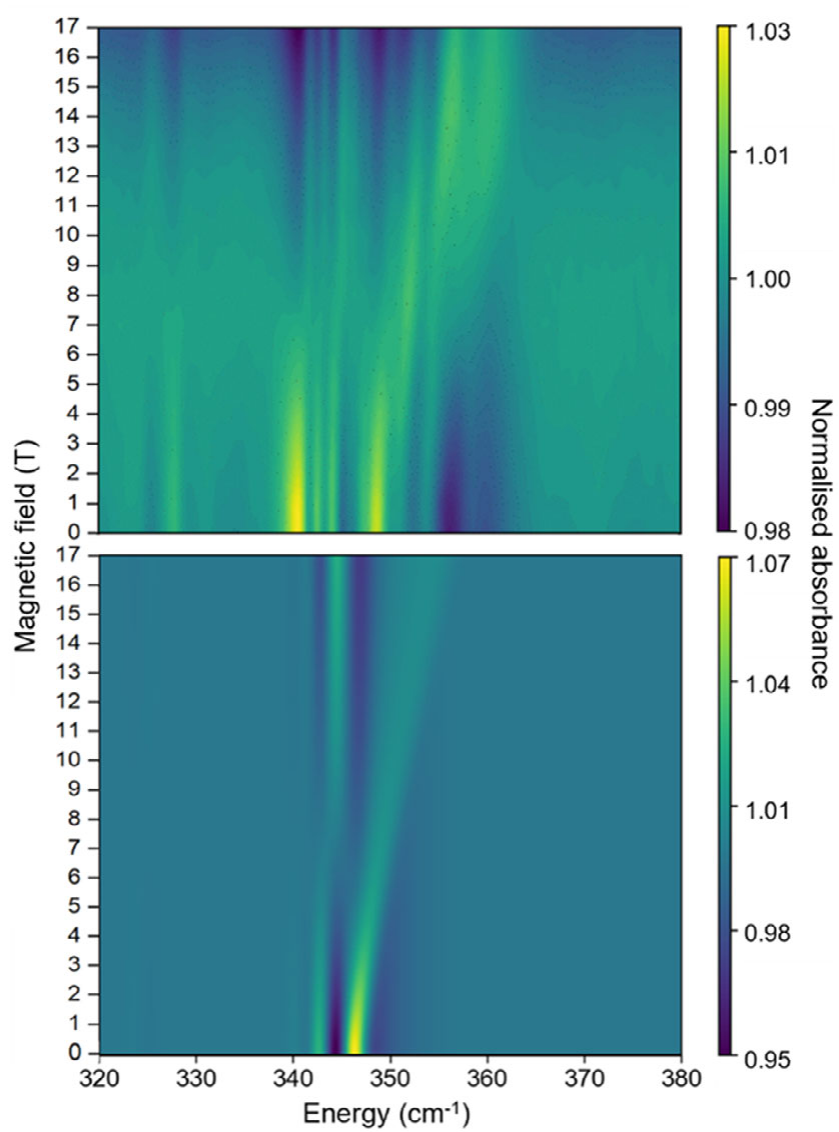
Experimental (top) and simulated (bottom) FIRMS heatmap of a powder sample of **1** in the 320–380 cm^−1^ energy region. The heatmap was generated from simulations using modes 67 and 68.

**FIGURE 4:**
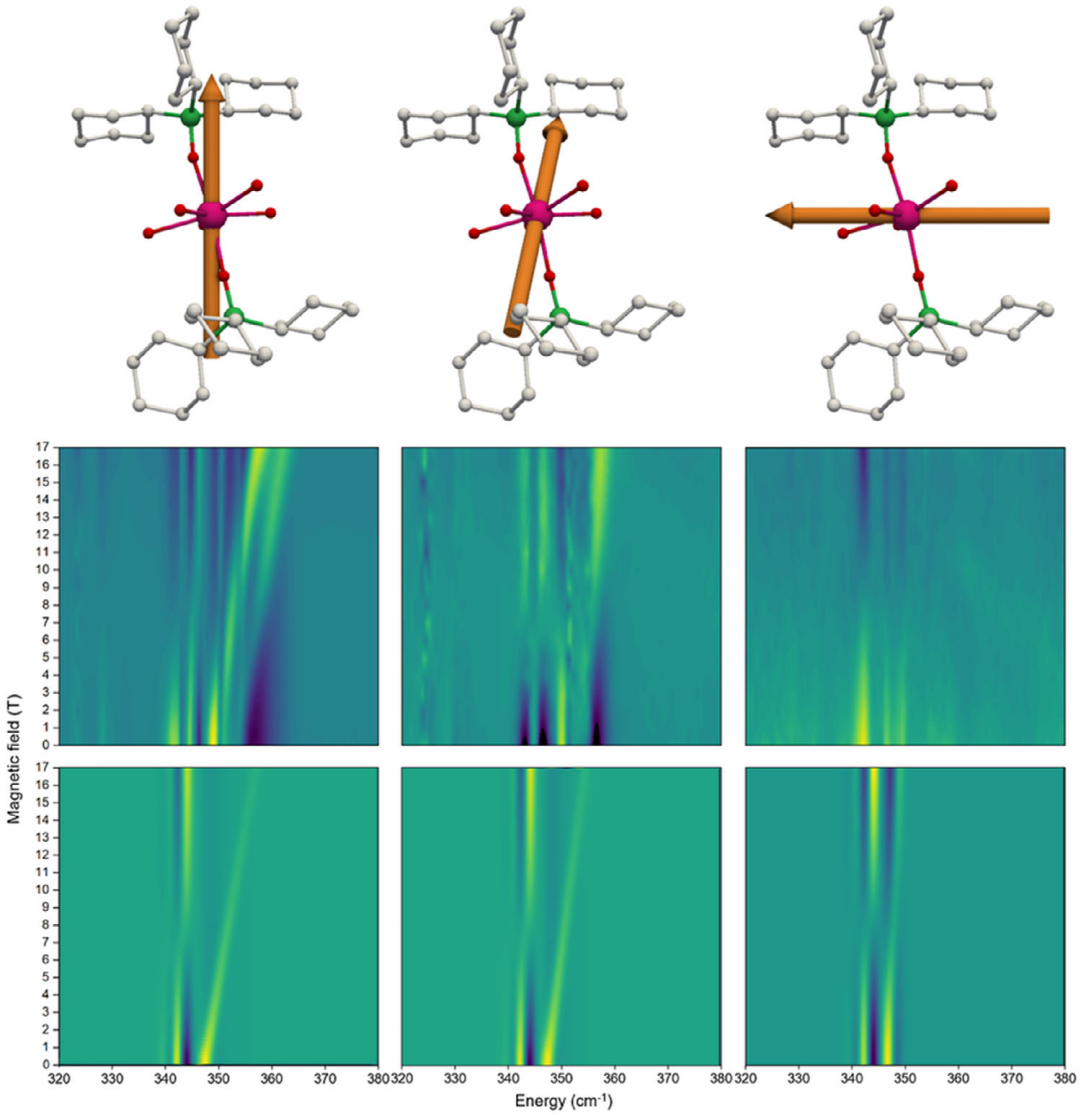
Molecular structure of the cationic unit of **1** (top), the main magnetic anisotropy axis is depicted by purple arrows and blue arrows represent magnetic field vectors. Experimental (middle) and simulated (bottom) FIRMS heatmaps of single‐crystal samples of **1‐*B1*** (left), **1‐*B2*** (middle) and **1‐*B3*** (right) in the 320–380 cm^−1^ energy range. The heatmaps were generated from simulations using modes 67 and 68.

To probe the spin‐phonon coupling, we have performed FIRMS measurements on a powder sample of **1** at 5 K with applied magnetic fields from 0 to 17.5 T. We distinguish magnetic absorptions from vibrational features by dividing the FIR transmission spectrum recorded at each magnetic field by the average of all spectra and plotting the intensity as a FIRMS heatmap (Figure 3); we find this distinguishes vibronic transitions more clearly compared to other common normalization schemes. As the measurements are conducted at 5 K, only the ground doublet is populated, and observable excitations will be either purely electronic or simultaneously electronic and vibrational (vibronic) in nature [20]. Furthermore, owing to the envelope effect of FIRMS transition intensities, vibrational modes with energies near to electronic transitions are enhanced [20]. Examination of the experimental data shows only one prominent feature between 340 cm^−1^ and 360 cm^−1^ (Figure 3, cf. Figures S20–S23), which is likely proximal to the magnetic dipole allowed ($\Delta \;m_{J}=\mp$1) electronic transition $m_{J}=\pm$15/2 to ±13/2. To probe the effect of anisotropy on spin‐phonon coupling we have also measured the FIRMS response of single‐crystal samples of **1** (see SI for details). We measure the FIRMS response for three orientations of the external magnetic field relative to the main magnetic anisotropy axis of [Dy(OPCy_3_)_2_(H_2_O)_5_]^3+^ in **1** (Figure S2): i) approximately parallel (∼19° off‐axis, **1‐*B1***, Figure 4, left); ii) glancing (∼36° off‐axis, **1‐*B2***, Figure 4, middle) and iii) approximately perpendicular (∼76° off‐axis, **1‐*B3***, Figure 4, right). The spectra show features in similar positions to that observed in the powder data, however, when the magnetic field is approximately parallel to the easy axis (**1‐*B1***) we observe the largest movement of the FIRMS transitions, while when it is approximately perpendicular to the easy axis (**1‐*B3***) we observe the least field‐dependence. This is commensurate with our expectations given the nature of the Zeeman splitting for near‐pure $m_{J}$ ground and first excited states that are quantised along the Cy_3_PO–Dy–OPCy_3_ axis.

To simulate the FIRMS spectra *ab initio*, we require a model of the vibrational modes: here, we optimize the geometry of the [Dy(OPCy_3_)_2_(H_2_O)_5_]^3+^ cation in the gas‐phase and obtain the harmonic normal modes of vibration using density‐functional theory (DFT) in Gaussian (see SI for details) [43]. The calculated IR spectrum agrees reasonably well with the experimental spectrum (Figure S4). A CASSCF‐SO calculation for the electronic structure using this geometry yields a similar first excitation energy of 432 cm^−1^ as we found for the isolated [Dy(OPCy_3_)_2_(H_2_O)_5_]^3+^ cation using the X‐ray structure (Table S3 cf. Table S1), which deviates by ca. 100 cm^−1^ from the experimental FIRMS data. Given the minor effect of using a gas‐phase optimized geometry, we suspected that this discrepancy came from omitting the solid‐state environment of **1** in the electronic structure calculation. Optimizing the geometry of the entire molecular crystal using periodic DFT with VASP [44, 45, 46, 47], then using an electrostatic embedding approach to simulate periodic crystalline electrostatic potential (see SI for details) [48], we performed a CASSCF‐SO calculation for [Dy(OPCy_3_)_2_(H_2_O)_5_]^3+^, which now includes the effects of counterions and solvent molecules on the electronic structure. These again give a $m_{J}=\pm$15/2 ground state, however, this calculation shows a significantly smaller energy gap to the first excited state of 356 cm^−1^ (Table S4). Thus, we have shifted the equilibrium energies of our gas‐phase optimized structure to match those of the solid‐state calculation to better represent the experiment, however, we continue to use the spin‐phonon coupling parameters from the DFT‐optimized gas‐phase model (see SI for details) [49]. Our FIRMS simulations utilize the *FIRMS_SIM* code [20], wherein owing to computational limitations we must select a sub‐set of vibrational modes for any given simulation. Preliminary simulations using low‐energy vibrational modes were unable to replicate the features observed in the experimental spectrum; we suspect this arises due to the envelope effect [20], where vibrational modes that are near‐resonant with electronic transitions tend to dominate the FIRMS spectrum, and not necessarily those with strong vibronic coupling. Hence, our simulations focus on modes 67 and 68 (341 and 344 cm^−1^, respectively,) as they replicate most of the notable features in the experimental powder spectrum between 320–380 cm^−1^ (Figure 3). The inclusion of mode 69 in the simulations dominates the spectrum with an additional feature around 360 cm^−1^ (Figure S5), so we are not convinced vibronic excitations are observed including this mode, however it could be that the mode energy is slightly over predicted. As these mode energies are a significant fraction of the experimental excitation, the experimentally observed features must include a purely electronic transition ($m_{J}=-$15/2 to −13/2) as well as intra‐Kramers vibronic transitions (excitation modes 67 or 68 along with the low‐energy $m_{J}=-$15/2 to +15/2 transition). These modes have vibronic coupling strengths (*S_j_*, Equation S3, Figure S6 and Table S5) of around 0.02 cm^−1^ and involve O–H stretches of the equatorial water molecules, which is weaker than the coupling for the observable intra‐Kramers modes in [Yb(trensal)] [20]. These latter vibronic features are intense here due to the envelope effect that reveals how intra‐Kramers signals are enhanced when the energy of the vibrational modes are similar to that of the CF energy gaps [20]. Nevertheless, these are not the most strongly coupled vibrational modes: mode 153 (1,008 cm^−1^) has the largest *S_j_* value of 0.16 cm^−1^ and corresponds to symmetric stretching of the oxygen atoms in the O–Dy–O axial bonds, while modes 95 and 97 (488 and 496 cm^−1^, respectively) have *S_j_* = 0.14 cm^−1^ and also involve distortion of the Dy–O bonds. However, owing to the envelope effect [20], there are no strong features in the FIRMS spectra at around 488 and 496 cm^−1^ corresponding to modes 95 and 97 (Figure S7).

Moving on to the single‐crystal FIRMS spectra of **1**, simulations using only modes 67 and 68 are in quite good agreement with the experimental spectra (Figure 4). Matching the experimental results, the largest field‐dependence is calculated when the magnetic field is roughly parallel to the main anisotropy axis (**1‐*B1***), and smallest when it is roughly perpendicular (**1‐*B3***). Examining the vibronic eigenstates involved in the transitions, at small magnetic field values the dominant transitions in all three cases have contributions from both intra‐Kramers vibronic and purely electronic transitions. As the strength of the magnetic field increases, the purely electronic transition becomes solely dominant from 5 T onwards for **1‐*B1***, illustrated by a large field‐dependence in the FIRMS spectrum. However for **1‐*B2*** and **1‐*B3*** when the field is glancing or near perpendicular, the transitions continue to be more mixed in character.

**FIGURE 5:**
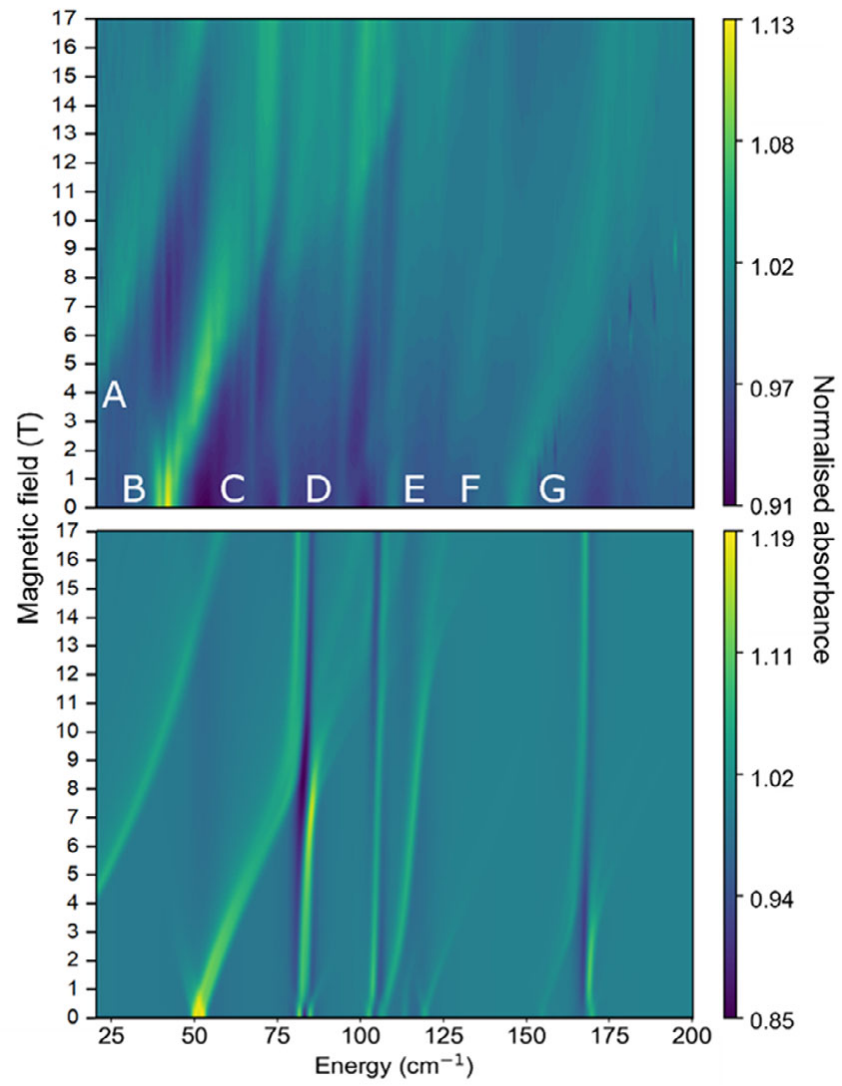
Experimental (top) and simulated (bottom) FIRMS heatmap of a powder sample of **2** in the 20–200 cm^−1^ energy range. The heatmap was generated from simulations using modes 13–16, 17–21, and 28–30.

The FIRMS response of complex **2**, in contrast to that of **1**, shows many low‐energy features between 20–200 cm^−1^ with clear field‐dependence (Figure 5, see Figure S24 for full field range). The strongest feature appears around 40 cm^−1^ at zero field, with others at 70, 100 and 150 cm^−1^. These field‐dependent features likely indicate magnetic transitions from the ground $m_{J}=\pm$1/2 state to excited states, but also likely involve vibronic coupling. As before, we obtain the vibrational modes for **2** by a gas‐phase DFT optimization and perform CASSCF‐SO calculations using the optimized geometry to obtain the static electronic structure as well as the spin‐phonon coupling (Table S6). The first electronic excited state predicted by CASSCF‐SO using the optimized structure of **2** is 49 cm^−1^, consistent with a strong FIRMS transition at 40 cm^−1^; thus, we continue with the optimized gas‐phase model in this case. Our best reproduction of the highly‐featured low‐energy region of the experimental FIRMS heatmap is produced by performing three different simulations including vibrational modes 13–16, 17–21 and 28–30 (81‐102, 103–112 and 165–167 cm^−1^), respectively and creating a composite heatmap that reproduces features **A**‐**G** from 20–200 cm^−1^ (Figure 5). The feature appearing at 25 cm^−1^ (**A**) appears in all simulations and we assign this to the EPR transition $m_{J}=-$1/2 to +1/2, as this appears to originate from 0 cm^−1^. Peak **B** at around 50 cm^−1^ that moves to higher energy with increasing magnetic field is indicative of a purely electronic transition ($m_{J}=-$1/2 to −3/2), but also exhibits a small feature moving to lower energy with increasing field, which suggests there is also a contribution from the electronically hot transition, $m_{J}=+$1/2 to +3/2. Another electronic transition is depicted by feature **G** that moves from around 150 cm^−1^ at 0 T to 180 cm^−1^ as the field increases to 17.5 T and can be simulated by including modes 28–30 (Figure S9). We assign this peak to the $m_{J}=-$1/2 to −5/2 transition due to it occurring at similar energy to that of the calculated value (147 cm^−1^, Table S6) and moving to higher energy with increasing magnetic field. We assign the peak at around 75 cm^−1^ (**C**) to an intra‐Kramers vibronic transition coupled to modes 13 and 14 (81 and 83 cm^−1^, Figure S10) and rule out an inter‐Kramers transition that would involve the electronic transition $m_{J}=\pm$1/2 to ±3/2 at ∼ 50 cm^−1^ coupled to modes 1–2 (28 cm^−1^), as this presents no intensity in this energy region of the simulated heatmap (Figure S11). A simulation involving modes 17–20 reproduces features between 100–125 cm^−1^ (Figure S12), allowing us to assign signal **D**, at around 100 cm^−1^, to an intra‐Kramers vibronic transition coupling to modes 17 and 18 (103 and 104 cm^−1^). Simulations including modes 20–21 replicate fainter features **E** and **F** (Figure S13), likely due to intra‐Kramers transitions coupled to these modes. The *S_j_* values of the vibrational modes in complex **2** are similar to those of **1** (Figure S14 and Table S7), the largest being modes 20 and 21 at 111 and 112 cm^−1^, both with values of 0.15 cm^−1^ and corresponding to bending motion of the Dy–N bonds. FIRMS spectra of complex **2** display coupling in the form of avoided crossings, similar to that observed for the erbium(III) analogue: [34] as feature **B** shifts to higher energy, it begins a series of avoided crossings with peaks **C**, **D** and **E** up to around 120 cm^−1^, which is well‐captured by our simulations. The first instance with **C** involves coupling of vibrations with the $m_{J}=\pm$1/2 to ±3/2 transitions and as the peak shifts with increasing magnetic field, it continues to couple with further vibrations. Another avoided crossing is present at feature **G**, which involves coupling of vibrations with the ground to second excited state $m_{J}=\pm$1/2 to ±5/2 transition.

The opposing anisotropy of complexes **1** and **2** is clearly revealed in the FIRMS spectra, where the FIRMS response of **2** displays many features in the low energy region (< 200 cm^−1^) compared to **1** which only presents one main feature at a higher energy (∼ 350 cm^−1^), despite the similar *S_j_* values of vibrational modes in both complexes. The spin‐phonon couplings in **2** appear in the spectra as avoided crossings, differing to **1** which exhibits strong vibronic transitions around the energy of the first electronic excitation due to the envelope effect, where the feature differs depending on the orientation of magnetic field relative to the easy‐axis.

## 3 Conclusion

By fusion of FIRMS experiments with *ab initio* calculations, we have investigated the spin‐phonon (vibronic) coupling in two dysprosium complexes. Our excellent simulations of powder and single‐crystal spectra allow us to have confidence in the underlying spin‐phonon coupling determined by the calculations. Our analysis of the vibronic coupling in an easy‐axis dysprosium(III) SMM compared to that of an easy‐plane dysprosium(III) non‐SMM, shows that this coupling is more extensive in the non‐SMM and shows evidence of vibronic avoided‐crossings. Importantly, single‐crystal FIRMS measurements confirm predictions from *ab initio* calculations on the nature of the magnetic anisotropy, and are hence powerful tools alongside single crystal magnetometry. In this work, FIRMS has enabled the direct probing of vibronic coupling in two dysprosium(III) complexes, revealing that vibronic features in the FIRMS spectra of the SMM are prevalent near energies of the electronic excitation, while the complex with smaller magnetic anisotropy shows much more substantial vibronic coupling. Modern research on the spin‐phonon coupling in magnetic molecules by both experimental and theoretical means will continue to facilitate the development of chemical strategies to reduce, or enhance, coupling.

## Conflicts of Interest

The authors declare no conflict of interest.

## Supporting information




**Supporting file 1**: The authors have cited additional references within the Supporting Information [14, 20, 35, 36, 39, 40, 43, 44, 45, 46, 47].
