# Supplementary material for: Measurement and Analysis of Vibronic Coupling in Two Dysprosium(III) Complexes of Opposite Magnetic Anisotropy
Source: Chemistry. 2025 Dec 12;32(4):e03558. doi: 10.1002/chem.202503558 (PMC12840834; doi:10.1002/chem.202503558)
Supplement: Supplementary file 1 — Supporting file 1: The authors have cited additional references within the Supporting Information [14, 20, 35, 36, 39, 40, 43, 44, 45, 46, 47]. [file CHEM-32-e03558-s001.pdf]

Supporting Information for

Measurement and analysis of vibronic coupling in two dysprosium(III) complexes of opposite magnetic anisotropy

Y. L. Whyatt,[1] J. Emerson-King,[1] G. F. S. Whitehead,[1] D. P. Mills,[1*] S. K. Langley,[2*] M. Ozerov[3*] and N. F. Chilton[1,4*]

[1] Department of Chemistry, The University of Manchester, Manchester, M13 9PL, UK

[2] Division of Chemistry, Manchester Metropolitan University, Manchester, M15 6BX, UK

[3] National High Magnetic Field Laboratory, Florida State University, Tallahassee, Florida, FL 32310, USA

[4] Research School of Chemistry, The Australian National University, Canberra, 2601, Australia

Synthesis

Compounds **1** and **2** were synthesised and purified by recrystallisation following published procedures.[1,2] The identities of **1** and **2** were confirmed by single crystal XRD experiments which were in agreement with the previously reported data.

Far-Infrared Magneto-Spectroscopy (FIRMS)

Far-IR measurements under applied magnetic fields were conducted at the National High Magnetic Field Laboratory (Tallahassee, USA) using a Bruker Vertex 80v spectrometer coupled to a 17.5 T vertical-bore superconducting magnet. The light-pipe optical probe was equipped with a Si bolometer detector, and both the detector and the sample were cooled with low-pressure helium gas to approximately 5 K. Eicosane was used to secure the powder and single-crystal samples at the 3 mm clear optical aperture of the sample holder. Transmission spectra were collected in the 10–720 cm^{-1} range (Figures S15–S19) with an instrumental resolution of 0.3 cm^{-1}. Samples of compound **1** were prepared in a glovebox under an argon atmosphere with strict exclusion of moisture and oxygen. Polypropylene tape was used to encapsulate and protect the sample during installation into the probe.

Ab initio Calculations

The Gaussian 09d[3] software was used to perform geometry optimisations and normal mode calculations on the crystal structures of **1** and **2** in the gas-phase, whilst solid-state calculations involved determining the CHELPG charges[4] of atoms in the primitive cell to represent the molecular electrostatic potential of the crystal. The PBE density functional and Grimme's D3 dispersion correction were employed.[5,6] The cc-pVTZ basis set was used for oxygen, nitrogen and phosphorus atoms, whilst the cc-pVDZ was used for carbon, silicon and hydrogen atoms.[7] In both cases, dysprosium was substituted for yttrium with an isotopic mass of 162.5 to aid self-consistent field

convergence, for which the Stuttgart RSC 1997 effective core potential (ECP) basis set was used for the 28 core electrons, with the remaining valence electrons being described by the corresponding valence basis set.

Geometry optimisation on the X-ray geometry of the primitive cell of **1** was performed using the Vienna Ab initio Simulation Package (VASP) 5.4.4 code,[8–11] atomic cores of each element were modelled with the projector-augmented wave (PAW) pseudopotentials[12,13] and a plane-wave cutoff energy of 800 eV determined by convergence testing. The Brillouin zone was sampled using the Γ-point. A convergence criteria of 10^{-8} eV for electronic energy and 10^{-3} eV A^{-1} for forces was employed. Phonon calculations on the optimised structure were then executed using the Phonopy code,[14,15] where force constants were determined for the 1×1×1 supercell.

The OpenMolcas[16,17] package was used to perform state-averaged complete active space self-consistent field spin-orbit (CASSCF-SO) calculations on the X-ray crystallographic and PBE-optimised structures of **1** and **2**, as well as on a single molecule of **1** embedded in the centre of a sphere (40 Å radius) of unit cells where remaining atoms are represented by the CHELPG charges previously calculated. The active space included nine 4f electrons in seven 4f orbitals of Dy^{III} and the 18 lowest lying sextet roots. Dysprosium atoms were modelled with the ANO-RCC-VTZP, oxygen and nitrogen atoms with ANO-RCC-VDZP and remaining atoms with the ANO-RCC-VDZ basis set.[18] The atomic-compact Cholesky decomposition (acCD) scheme was used to treat two-electron integrals.[19] After CASSCF optimisation, the states were mixed by spin-orbit coupling using the RASSI module and the electronic structures projected onto a crystal field Hamiltonian using SINGLE_ANISO.[20] Spin-phonon couplings were also calculated in OpenMolcas, where crystal field parameters were determined by calculating nonadiabatic couplings and analytical gradients using a linear vibronic coupling (LVC) method.[21]

FIRMS simulations were performed using the *FIRMS_SIM* code developed by Kragskow and Chilton (specified in Ref. [22]), which enabled simulation of normalised FIRMS absorbance spectra in the form of heatmaps.

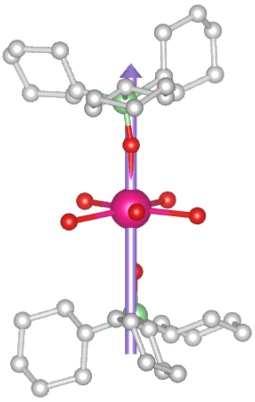

Figure S1. Structure of **1** showing the g_z vector of the ground state determined from CASSCF-SO calculations.

Table S1. Electronic structure of the crystal geometry using a single cation of **1**.

**Energy (cm^{-1})**	**Energy (K)**	g_x	g_y	g_z	**θ (°)**	**Wavefunction**	**<J_z>**
0	0	0.00	0.00	19.98	--	100% |±15/2⟩	± 7.50
451	649	0.00	0.00	17.06	0.60	100% |±13/2⟩	± 6.50
808	1163	0.03	0.03	14.28	1.73	99% |±11/2⟩	± 5.48
1031	1484	0.92	2.25	10.78	14.17	85% |±9/2⟩	± 4.04
1057	1520	0.58	2.18	16.71	88.73	47% |∓1/2⟩ + 28% |±3/2⟩	± 0.33
1113	1601	2.84	8.34	9.96	87.33	42% |±7/2⟩ + 21% |±3/2⟩ + 15% |∓5/2⟩	± 1.19
1155	1662	1.16	2.33	17.24	78.59	25% |±5/2⟩ + 24% |±7/2⟩ + 17% |±3/2⟩	± 1.34
1222	1758	0.09	0.28	19.21	73.11	34% |±5/2⟩ + 22% |±7/2⟩ + 20% |±3/2⟩	± 2.06

Table S2. Electronic structure of the crystal geometry using a single molecule of **2**.

**Energy (cm^{-1})**	**Energy (K)**	g_x	g_y	g_z	**θ (°)**	**Wavefunction**	**<J_z>**
0	0	1.39	10.33	10.99	--	99% |±1/2⟩	± 0.50
51	73	0.32	0.34	4.20	89.42	99% |±3/2⟩	± 1.52
155	224	1.48	1.51	6.83	89.96	98% |±5/2⟩	± 2.48
321	462	1.47	1.48	9.55	89.97	98% |±7/2⟩	± 3.48
548	788	0.00	0.00	12.28	89.97	98% |±9/2⟩	± 4.50
833	1199	0.01	0.01	14.79	89.97	99% |±11/2⟩	± 5.47
1140	1640	0.01	0.01	17.31	89.98	99% |±13/2⟩	± 6.46
1417	2038	0.00	0.00	19.87	89.96	99% |±15/2⟩	± 7.47

To measure the FIRMS spectra of single-crystal samples with the external magnetic field in three main directions (Figure S2). **1-*B1*** and **1-*B3*** were both measured in the Voigt geometry, with the magnetic field applied either perpendicular or parallel to the plane of the sample, respectively, and perpendicular to the light propagation vector. Whereas for **1-*B2***, the measurement was in the Faraday geometry, where the magnetic field was applied perpendicular to the plane of the sample with unpolarised light propagating in the same direction. Intensities were calculated according to reference [23].

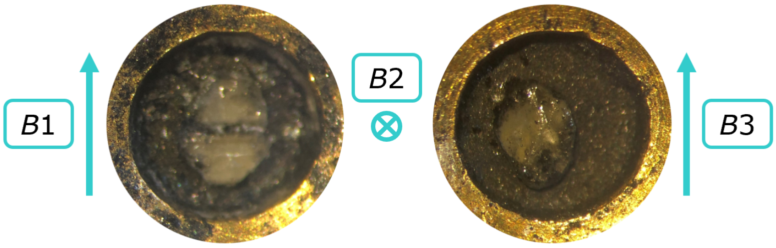

Figure S2. Images of single-crystals of **1** in the sample holder. The three magnetic field directions relative to the crystals are shown in turquoise. No image was taken for *B2*, but the magnetic field was aligned perpendicular to the plane of the single-crystal.

Crystal face indexing was carried out on a single-crystal of **1** to assign the Miller indices of each face (Figure S3). The unit cell of **1** was then aligned with the crystal to determine the Miller planes in the molecular frame, which enabled the magnetic field vectors to be determined and inputted into the *FIRMS_SIM* code.

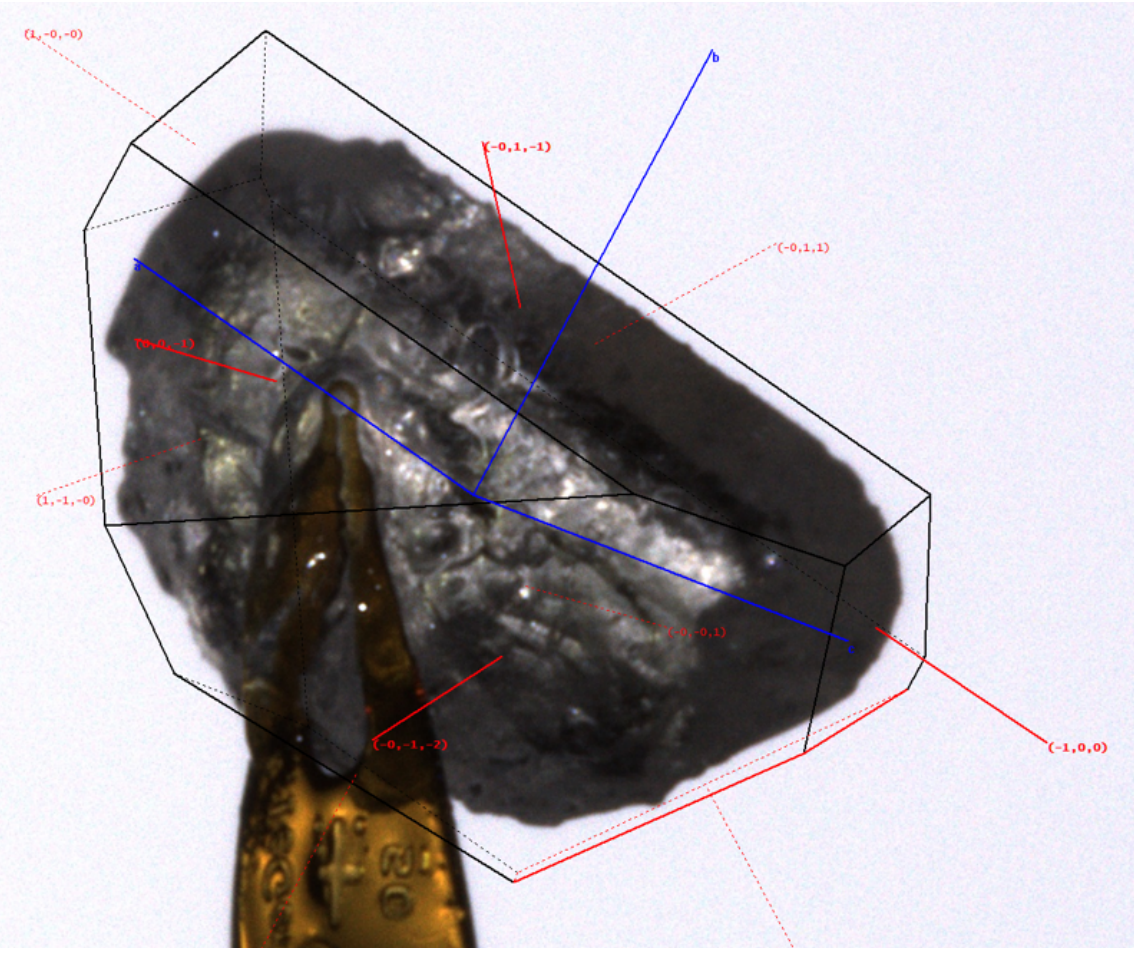

Figure S3. A single crystal of **1** mounted on a goniometer head. The red overlay represents the crystal faces with the miller indices assigned, and the blue overlay represents the unit cell axes.

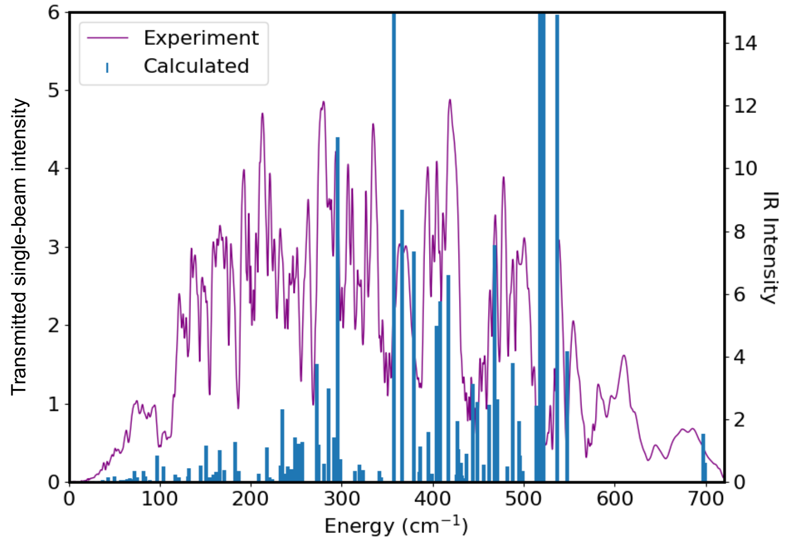

Figure S4. Experimental (purple) and calculated (blue) IR spectra of a powder sample of **1** in the 0-720 cm^{-1} energy range.

Table S3. Electronic structure of the gas-phase optimised geometry of **1**.

Energy (cm^{-1})	Energy (K)	g_x	g_y	g_z	θ (°)	Wavefunction	$<J_z>$
0	0	0.00	0.00	19.98	--	100% |±15/2⟩	± 7.50
432	622	0.02	0.02	17.05	0.90	99% |±13/2⟩	± 6.48
747	1075	0.40	0.57	14.03	2.46	97% |±11/2⟩	± 5.37
891	1281	1.90	2.50	15.37	89.48	44% |±1/2⟩ + 17% |∓3/2⟩ + 17% |±9/2⟩	± 0.73
928	1336	0.99	3.01	11.79	71.64	40% |±9/2⟩ + 19% |±1/2⟩ + 12% |∓1/2⟩	± 1.91
957	1377	1.43	4.50	9.30	66.48	38% |±3/2⟩ + 26% |±9/2⟩	± 1.54
985	1417	1.30	3.77	11.37	70.46	37% |±5/2⟩ + 19% |±7/2⟩	± 1.47
1026	1476	2.08	4.83	14.11	73.13	55% |±7/2⟩ + 18% |∓5/2⟩ + 16% |±5/2⟩	± 1.81

Table S4. Electronic structure of the solid-state optimised geometry of **1**.

Energy (cm^{-1})	Energy (K)	g_x	g_y	g_z	θ (°)	Wavefunction	<J_z>
0	0	0.00	0.00	19.98	--	100% |±15/2⟩	± 7.50
356	512	0.01	0.01	17.10	0.56	100% |±13/2⟩	± 6.50
617	888	0.16	0.33	14.11	3.45	96% |±11/2⟩	± 5.37
675	972	0.18	0.56	18.89	88.03	38% |±1/2⟩ + 26% |∓1/2⟩ + 16% |±3/2⟩	± 0.26
734	1056	2.90	3.44	13.03	74.96	27% |±9/2⟩ + 19% |±5/2⟩ + 18% |±3/2⟩	± 1.74
773	1111	2.84	5.44	9.37	68.86	52% |±9/2⟩ + 15% |±3/2⟩	± 2.27
805	1158	1.39	2.78	16.30	67.83	37% |±7/2⟩ + 35% |±5/2⟩ + 11% |±1/2⟩	± 2.40
873	1256	0.19	0.32	18.82	65.41	42% |±7/2⟩ + 27% |±5/2⟩ + 12% |±9/2⟩	± 2.92

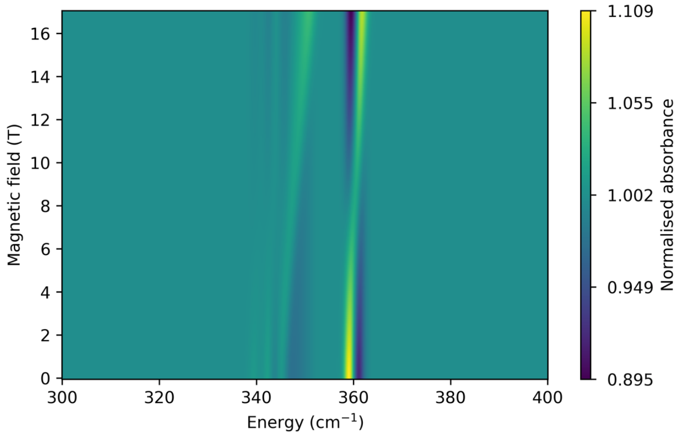

Figure S5. Simulated FIRMS heatmap of a powder sample of **1** in the 300-400 cm^{-1} energy range using modes 67-69 (341-357 cm^{-1}).

To evaluate how strongly each vibrational mode of the molecules couples, the vibronic coupling strength (S_j) can be calculated using Equation S1,[22,24] where Q_j is the normal mode displacement and B_q^k are CFPs in Wybourne notation, which are linear combinations of the CFPs expressed in Stevens notation, B_k^q.[25]

$$S_j = \sqrt{\frac{1}{3}\sum_k \frac{1}{2k+1}\sum_{q=-k}^{k}\left|\left(\frac{\partial B_q^k}{\partial Q_j}\right)_{\text{eq}}\right|^2} \quad \text{(S1)}$$

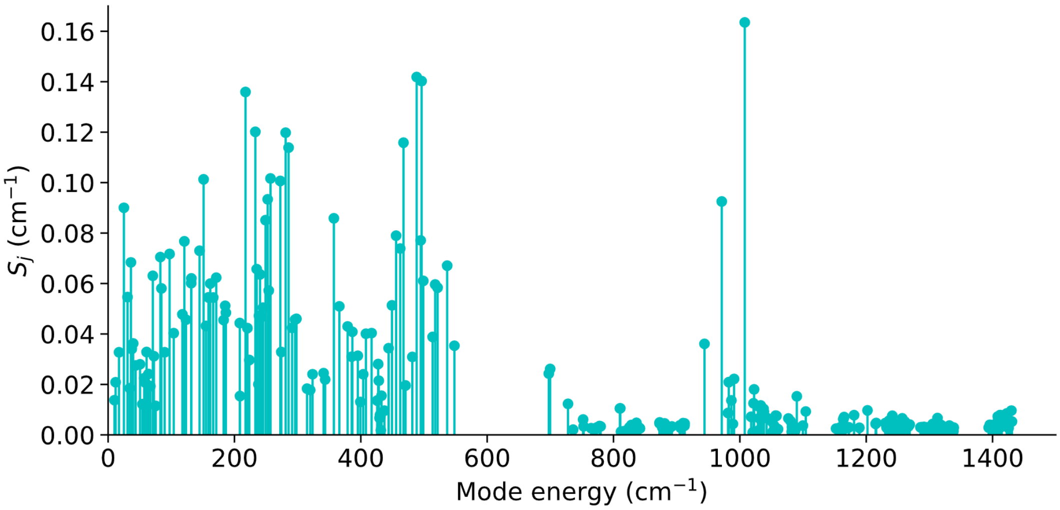

Figure S6. Vibrational coupling strength (S_j) of each vibrational mode of **1** in the range of 0-1500 cm^{-1}.

Table S5. Vibrational mode energies (cm^{-1}) and S_j (cm^{-1}) of **1** from gas-phase optimisation.

Mode	Energy (cm^{-1})	S_j (cm^{-1})	Mode	Energy (cm^{-1})	S_j (cm^{-1})	Mode	Energy (cm^{-1})	S_j (cm^{-1})
1	10	1.38E-02	121	828	3.93E-03	241	1330	3.39E-03
2	12	2.09E-02	122	833	2.54E-03	242	1331	3.25E-03
3	17	3.27E-02	123	836	1.26E-03	243	1332	3.68E-03
4	25	9.00E-02	124	836	4.75E-03	244	1336	2.95E-03
5	31	5.46E-02	125	837	2.46E-03	245	1336	2.63E-03
6	34	1.85E-02	126	840	3.00E-03	246	1337	9.79E-04
7	36	6.84E-02	127	842	2.57E-03	247	1337	2.10E-03
8	37	3.40E-02	128	873	4.60E-03	248	1338	1.48E-03
9	40	3.62E-02	129	873	4.97E-03	249	1339	2.86E-03
10	44	2.75E-02	130	875	4.20E-03	250	1394	2.87E-03
11	51	2.80E-02	131	877	2.92E-03	251	1395	3.98E-03
12	54	1.22E-02	132	878	3.69E-03	252	1398	3.30E-03
13	57	2.25E-02	133	878	3.77E-03	253	1398	3.91E-03
14	59	2.07E-02	134	879	3.36E-03	254	1399	2.17E-03
15	61	3.29E-02	135	881	4.55E-03	255	1401	2.33E-03
16	64	2.41E-02	136	883	3.79E-03	256	1402	1.77E-03
17	67	1.93E-02	137	886	2.42E-03	257	1402	3.55E-03
18	71	6.31E-02	138	888	2.34E-03	258	1403	1.15E-03
19	73	3.12E-02	139	892	3.36E-03	259	1404	2.10E-03
20	75	1.15E-02	140	905	3.72E-03	260	1404	2.17E-03
21	83	7.05E-02	141	906	2.83E-03	261	1405	3.08E-03
22	84	5.80E-02	142	910	4.40E-03	262	1405	7.11E-04
23	90	3.28E-02	143	910	4.64E-03	263	1405	1.50E-03
24	97	7.17E-02	144	912	3.62E-03	264	1406	8.52E-04
25	104	4.03E-02	145	913	4.55E-03	265	1408	5.06E-03

26	118	4.78E-02	146	944	3.61E-02	266	1408	7.27E-03
27	121	7.67E-02	147	971	9.25E-02	267	1409	2.63E-03
28	123	4.57E-02	148	981	8.66E-03	268	1411	5.83E-03
29	132	6.01E-02	149	982	2.09E-02	269	1411	3.81E-03
30	132	6.20E-02	150	987	1.37E-02	270	1412	7.82E-03
31	145	7.30E-02	151	989	4.33E-03	271	1413	7.85E-03
32	151	1.01E-01	152	991	2.22E-02	272	1413	3.12E-03
33	155	4.32E-02	153	1008	1.64E-01	273	1414	5.03E-03
34	159	5.45E-02	154	1018	7.18E-03	274	1423	8.48E-03
35	162	6.00E-02	155	1020	9.59E-04	275	1423	8.02E-03
36	166	5.44E-02	156	1022	1.25E-02	276	1425	2.71E-03
37	171	6.23E-02	157	1023	1.81E-02	277	1428	5.16E-03
38	183	4.55E-02	158	1029	1.14E-02	278	1430	9.62E-03
39	185	5.12E-02	159	1030	1.80E-03	279	1431	5.29E-03
40	186	4.84E-02	160	1031	6.60E-03	280	1544	4.37E-02
41	208	4.43E-02	161	1033	1.17E-02	281	1554	4.06E-02
42	209	1.54E-02	162	1036	6.52E-03	282	1557	4.38E-02
43	217	1.36E-01	163	1037	1.03E-02	283	1575	3.98E-02
44	221	4.23E-02	164	1038	9.25E-03	284	1579	3.84E-02
45	224	2.97E-02	165	1042	6.77E-03	285	2822	9.75E-03
46	233	1.20E-01	166	1051	5.21E-03	286	2837	7.90E-03
47	235	6.57E-02	167	1052	1.17E-03	287	2880	6.97E-03
48	238	2.01E-02	168	1055	3.44E-03	288	2907	2.38E-03
49	239	4.72E-02	169	1057	7.66E-03	289	2908	4.97E-03
50	240	6.36E-02	170	1058	7.54E-03	290	2908	4.40E-03
51	243	4.67E-02	171	1060	2.25E-03	291	2912	7.81E-03
52	245	5.04E-02	172	1077	6.52E-03	292	2923	6.47E-03
53	249	8.51E-02	173	1080	5.42E-03	293	2923	4.68E-03
54	253	9.34E-02	174	1082	1.21E-03	294	2927	4.11E-03
55	254	5.72E-02	175	1082	3.02E-03	295	2929	3.50E-03
56	257	1.02E-01	176	1083	3.38E-03	296	2930	4.29E-03
57	273	1.01E-01	177	1083	3.90E-03	297	2943	1.35E-03
58	274	3.29E-02	178	1087	1.53E-03	298	2944	2.76E-03
59	281	1.20E-01	179	1088	2.93E-03	299	2948	1.84E-03
60	286	1.14E-01	180	1089	2.58E-03	300	2949	2.32E-03
61	291	4.25E-02	181	1090	1.53E-02	301	2953	1.32E-03
62	296	4.57E-02	182	1100	3.70E-03	302	2957	1.34E-03
63	298	4.60E-02	183	1104	9.27E-03	303	2959	1.33E-03
64	315	1.83E-02	184	1152	2.50E-03	304	2959	1.81E-03
65	320	1.78E-02	185	1155	2.02E-03	305	2962	1.18E-03
66	324	2.40E-02	186	1159	2.99E-03	306	2963	7.22E-04
67	341	2.45E-02	187	1160	1.01E-03	307	2964	1.91E-03
68	344	2.19E-02	188	1164	6.29E-03	308	2968	1.76E-03
69	357	8.59E-02	189	1164	6.17E-03	309	2969	1.40E-03
70	366	5.10E-02	190	1165	7.05E-03	310	2971	4.00E-04

71	379	4.30E-02	191	1167	2.55E-03	311	2971	8.16E-04
72	386	3.10E-02	192	1173	2.98E-03	312	2974	9.15E-04
73	387	4.09E-02	193	1181	7.81E-03	313	2975	1.34E-03
74	395	3.13E-02	194	1189	2.83E-03	314	2975	1.16E-03
75	399	1.31E-02	195	1202	9.72E-03	315	2979	7.75E-04
76	404	2.40E-02	196	1215	4.34E-03	316	2980	9.75E-04
77	408	4.01E-02	197	1216	4.70E-03	317	2981	5.24E-04
78	417	4.04E-02	198	1230	4.65E-03	318	2981	7.63E-04
79	426	1.36E-02	199	1232	2.88E-03	319	2984	1.45E-03
80	427	2.81E-02	200	1233	4.23E-03	320	2984	3.96E-03
81	428	2.15E-02	201	1235	1.93E-03	321	2989	1.61E-03
82	430	6.65E-03	202	1236	5.60E-03	322	2993	3.73E-03
83	430	7.92E-03	203	1238	3.49E-03	323	2993	3.75E-03
84	431	7.40E-03	204	1241	7.56E-03	324	2998	3.08E-03
85	432	1.87E-03	205	1242	5.01E-03	325	3017	2.46E-03
86	433	1.55E-02	206	1245	3.90E-03	326	3018	1.77E-03
87	437	9.59E-03	207	1247	3.23E-03	327	3019	2.30E-03
88	444	3.44E-02	208	1251	2.28E-03	328	3019	3.14E-03
89	449	5.13E-02	209	1251	1.83E-03	329	3021	2.13E-03
90	456	7.90E-02	210	1255	2.64E-03	330	3021	1.11E-03
91	462	7.39E-02	211	1256	4.85E-03	331	3022	5.38E-04
92	468	1.16E-01	212	1257	6.55E-03	332	3025	2.20E-03
93	471	1.96E-02	213	1259	3.74E-03	333	3026	1.52E-03
94	482	3.09E-02	214	1261	5.26E-03	334	3027	2.00E-03
95	488	1.42E-01	215	1262	1.88E-03	335	3032	2.45E-03
96	495	7.71E-02	216	1265	3.55E-03	336	3033	1.46E-03
97	496	1.40E-01	217	1267	3.32E-03	337	3034	1.67E-03
98	499	6.10E-02	218	1268	3.95E-03	338	3035	1.97E-03
99	514	3.88E-02	219	1269	4.10E-03	339	3035	1.87E-03
100	518	5.96E-02	220	1286	2.98E-03	340	3036	1.91E-03
101	522	5.83E-02	221	1288	3.03E-03	341	3037	1.02E-03
102	537	6.71E-02	222	1292	3.16E-03	342	3038	5.51E-04
103	548	3.53E-02	223	1293	2.37E-03	343	3040	1.09E-03
104	698	2.43E-02	224	1296	2.91E-03	344	3041	1.39E-03
105	700	2.62E-02	225	1297	2.76E-03	345	3041	1.33E-03
106	728	1.23E-02	226	1304	1.22E-03	346	3041	1.38E-03
107	736	2.05E-03	227	1304	8.19E-04	347	3042	1.09E-03
108	752	6.07E-03	228	1305	4.33E-03	348	3043	8.66E-04
109	753	3.37E-03	229	1308	1.79E-03	349	3043	4.55E-04
110	765	2.66E-03	230	1308	1.87E-03	350	3043	1.17E-03
111	769	2.02E-03	231	1309	2.55E-03	351	3537	2.93E-02
112	773	2.62E-03	232	1309	3.68E-03	352	3543	4.29E-02
113	774	2.94E-03	233	1310	2.10E-03	353	3581	3.90E-02
114	778	3.51E-03	234	1312	3.38E-03	354	3611	3.60E-02
115	780	3.36E-03	235	1313	6.68E-03	355	3619	8.59E-03

116	810	1.05E-02	236	1313	1.38E-03	356	3647	3.28E-02
117	811	1.05E-02	237	1318	3.94E-03	357	3659	6.94E-03
118	812	1.28E-03	238	1326	1.36E-03	358	3686	1.54E-02
119	816	1.43E-03	239	1326	1.20E-03	359	3698	6.67E-03
120	825	3.01E-03	240	1328	3.15E-03	360	3731	4.62E-03

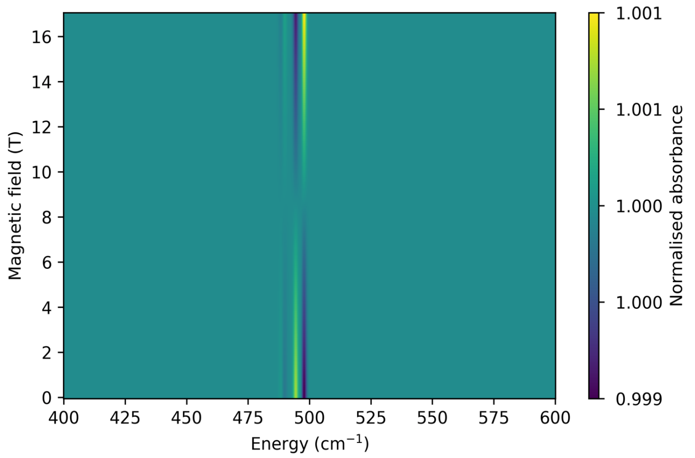

Figure S7. Simulated FIRMS heatmap of a powder sample of **1** in the 400-600 cm^{-1} energy range using modes 95-97 (488-496 cm^{-1}).

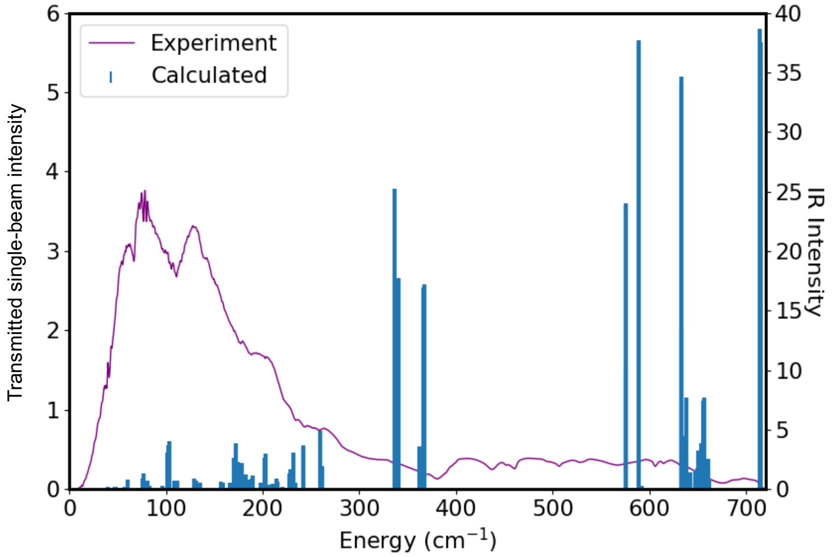

Figure S8. Experimental (purple) and calculated (blue) IR spectra of a powder sample of **2** in the 0-720 cm^{-1} energy range.

Table S6. Electronic structure of the gas-phase optimised geometry of **2**.

**Energy (cm^{-1})**	**Energy (K)**	g_x	g_y	g_z	**θ (°)**	**Wavefunction**	**<J_z>**
0	0	1.39	10.62	10.69	--	99% |±1/2⟩	± 0.51
49	70	0.03	0.04	4.22	89.92	99% |±3/2⟩	± 1.53
147	212	1.83	1.84	6.78	90.00	97% |±5/2⟩	± 2.47
304	438	1.81	1.81	9.48	90.00	97% |±7/2⟩	± 3.46
515	741	0.00	0.00	12.23	89.99	98% |±9/2⟩	± 4.49
778	1120	0.01	0.01	14.74	89.99	98% |±11/2⟩	± 5.45
1062	1528	0.02	0.02	17.28	89.99	98% |±13/2⟩	± 6.45
1338	1925	0.00	0.00	19.86	90.00	99% |±15/2⟩	± 7.47

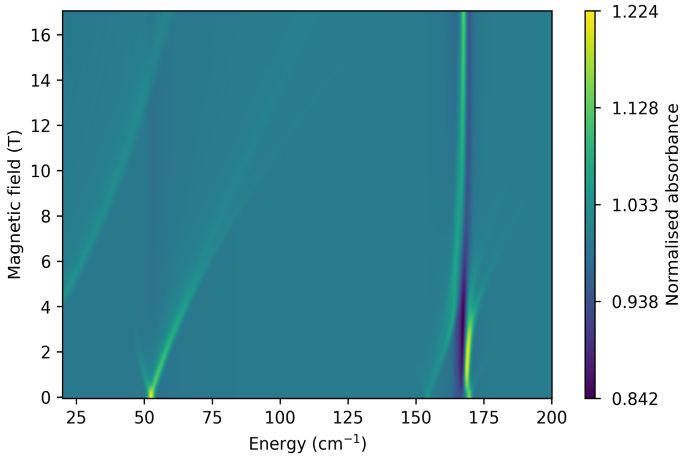

Figure S9. Simulated FIRMS heatmap of **2** in the 20-200 cm^{-1} energy range using modes 28-30 (165-167 cm^{-1}).

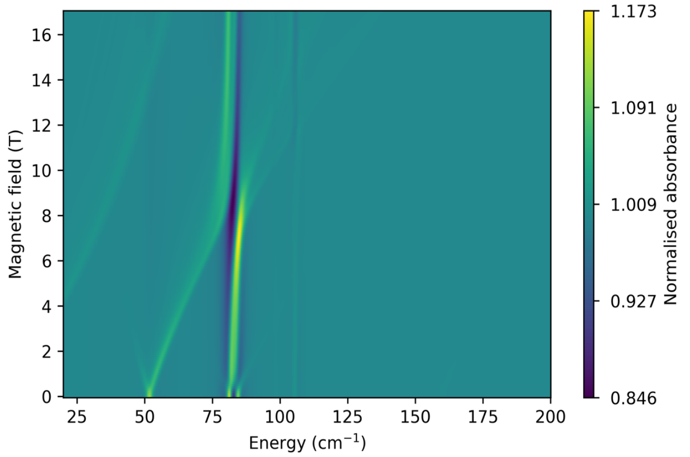

Figure S10. Simulated FIRMS heatmap of **2** in the 20-200 cm^{-1} energy range using modes 13-16 (81-102 cm^{-1}).

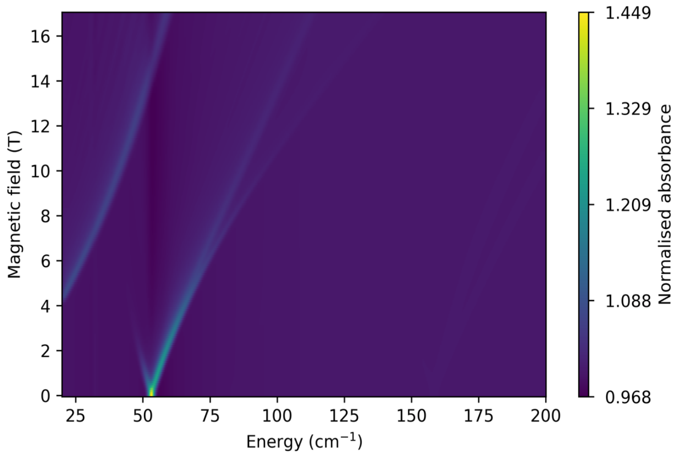

Figure S11. Simulated FIRMS heatmap of **2** in the 20-200 cm^{-1} energy range using modes 1-2 (28 cm^{-1}).

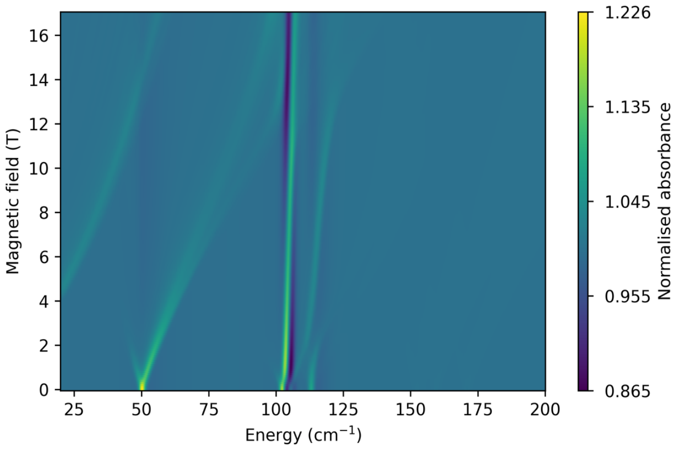

Figure S12. Simulated FIRMS heatmap of **2** in the 20-200 cm^{-1} energy range using modes 17-20 (103-111 cm^{-1}).

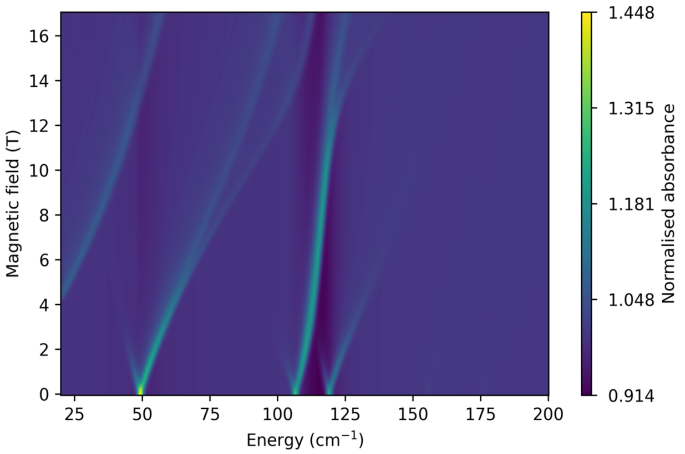

Figure S13. Simulated FIRMS heatmap of **2** in the 20-200 cm^{-1} energy range using modes 20-21 (111-112 cm^{-1}).

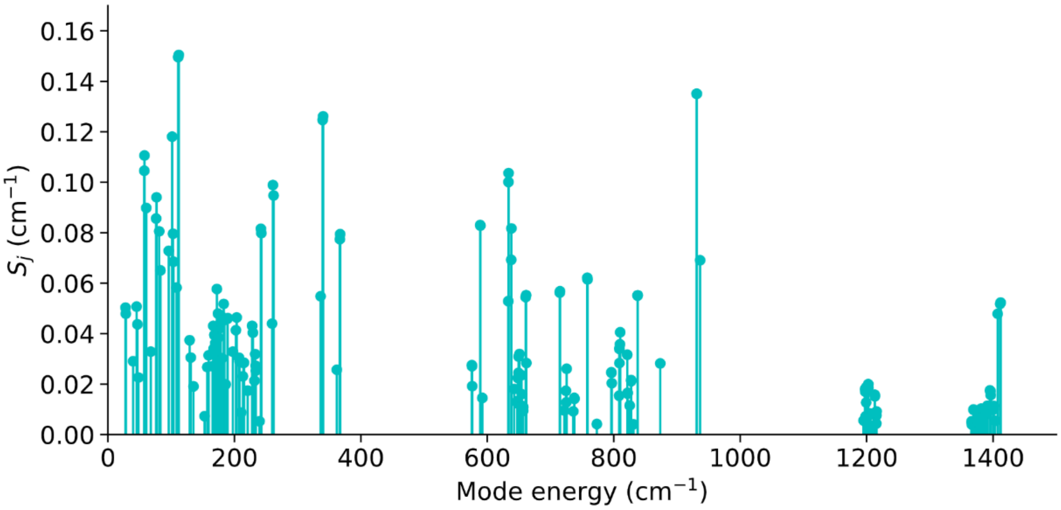

Figure S14. Vibrational coupling strength (S_j) of each vibrational mode of **2** in the range of 0–1500 cm^{-1}.

Table S7. Vibrational mode energies (cm^{-1}) and S_j (cm^{-1}) of **2** from gas-phase optimisation.

Mode	Energy (cm^{-1})	S_j (cm^{-1})	Mode	Energy (cm^{-1})	S_j (cm^{-1})	Mode	Energy (cm^{-1})	S_j (cm^{-1})
1	28	5.03E-02	121	634	1.04E-01	241	1375	3.95E-03
2	28	4.79E-02	122	638	6.93E-02	242	1375	5.35E-03
3	40	2.91E-02	123	638	8.17E-02	243	1376	4.91E-03
4	45	5.07E-02	124	642	1.81E-02	244	1377	6.40E-03
5	47	4.37E-02	125	647	1.33E-02	245	1377	6.73E-03
6	48	2.26E-02	126	648	1.23E-02	246	1380	2.18E-03
7	57	1.05E-01	127	648	2.24E-02	247	1381	1.03E-02
8	58	1.11E-01	128	649	2.45E-02	248	1381	9.83E-03
9	61	8.98E-02	129	650	3.09E-02	249	1385	8.59E-03
10	68	3.29E-02	130	651	3.19E-02	250	1386	9.65E-03
11	76	8.56E-02	131	654	1.15E-02	251	1386	8.61E-03
12	77	9.40E-02	132	654	2.35E-02	252	1387	8.31E-03
13	81	8.05E-02	133	654	1.60E-02	253	1387	7.51E-03
14	83	6.51E-02	134	656	1.13E-02	254	1388	1.00E-02
15	96	7.28E-02	135	657	9.40E-03	255	1391	1.14E-02
16	102	1.18E-01	136	657	1.09E-02	256	1391	1.13E-02
17	103	7.96E-02	137	661	5.45E-02	257	1395	1.75E-02
18	104	6.85E-02	138	661	5.52E-02	258	1396	1.72E-02
19	108	5.82E-02	139	662	2.83E-02	259	1396	1.57E-02
20	111	1.50E-01	140	715	5.63E-02	260	1400	1.03E-02
21	112	1.50E-01	141	715	5.68E-02	261	1400	9.88E-03
22	129	3.74E-02	142	722	9.54E-03	262	1401	1.14E-02
23	131	3.05E-02	143	724	1.73E-02	263	1403	5.47E-03
24	135	1.91E-02	144	725	1.28E-02	264	1407	4.79E-02
25	153	7.31E-03	145	725	2.61E-02	265	1411	5.18E-02

26	157	2.67E-02	146	736	9.21E-03	266	1412	5.23E-02
27	159	3.14E-02	147	738	1.45E-02	267	2916	2.67E-02
28	165	2.71E-02	148	738	1.42E-02	268	2916	2.68E-02
29	167	4.31E-02	149	758	6.21E-02	269	2920	2.15E-02
30	167	3.41E-02	150	759	6.15E-02	270	2961	2.72E-03
31	168	3.95E-02	151	773	4.18E-03	271	2961	2.63E-03
32	170	3.63E-02	152	796	2.47E-02	272	2962	1.00E-03
33	172	5.77E-02	153	796	2.45E-02	273	2962	2.64E-03
34	174	4.80E-02	154	797	2.04E-02	274	2962	2.67E-03
35	175	3.84E-02	155	808	1.54E-02	275	2963	2.83E-03
36	176	4.21E-02	156	809	3.40E-02	276	2963	2.08E-03
37	178	4.73E-02	157	809	2.83E-02	277	2963	1.59E-03
38	179	4.43E-02	158	810	3.58E-02	278	2964	1.62E-03
39	180	3.03E-02	159	810	4.05E-02	279	2967	2.87E-03
40	183	5.18E-02	160	821	3.17E-02	280	2967	2.89E-03
41	184	4.55E-02	161	822	1.62E-02	281	2967	2.60E-03
42	186	1.99E-02	162	822	1.66E-02	282	2971	1.71E-03
43	189	4.59E-02	163	825	1.15E-02	283	2972	1.56E-03
44	189	4.62E-02	164	828	2.16E-02	284	2972	1.69E-03
45	197	3.29E-02	165	828	2.12E-02	285	3003	1.91E-02
46	202	4.14E-02	166	831	4.06E-03	286	3004	1.90E-02
47	204	4.64E-02	167	838	5.51E-02	287	3006	2.43E-02
48	207	3.05E-02	168	838	5.52E-02	288	3036	1.85E-02
49	208	2.90E-02	169	874	2.82E-02	289	3036	1.87E-02
50	211	8.76E-03	170	931	1.35E-01	290	3037	1.02E-02
51	213	2.31E-02	171	931	1.35E-01	291	3051	1.92E-03
52	214	2.84E-02	172	937	6.91E-02	292	3051	2.02E-03
53	215	2.85E-02	173	1195	5.59E-03	293	3052	1.07E-03
54	221	1.74E-02	174	1197	1.80E-02	294	3054	1.16E-03
55	228	4.31E-02	175	1198	1.70E-02	295	3054	1.20E-03
56	229	4.04E-02	176	1198	7.26E-03	296	3054	8.58E-04
57	232	2.14E-02	177	1199	1.27E-02	297	3055	4.06E-03
58	233	3.19E-02	178	1199	1.88E-02	298	3055	3.87E-03
59	233	2.73E-02	179	1202	3.88E-03	299	3056	3.96E-03
60	234	2.53E-02	180	1203	2.00E-02	300	3057	4.91E-04
61	240	5.30E-03	181	1203	1.85E-02	301	3057	5.54E-04
62	242	8.16E-02	182	1203	2.52E-03	302	3058	2.88E-03
63	243	7.98E-02	183	1205	1.30E-03	303	3060	6.74E-04
64	260	4.40E-02	184	1205	8.31E-03	304	3060	4.06E-04
65	261	9.89E-02	185	1206	7.92E-03	305	3060	1.18E-03
66	262	9.47E-02	186	1213	1.58E-02	306	3061	1.87E-03
67	336	5.48E-02	187	1213	1.52E-02	307	3061	1.67E-03
68	339	1.25E-01	188	1215	4.36E-03	308	3061	1.56E-03
69	340	1.26E-01	189	1215	7.58E-03	309	3062	1.76E-03
70	362	2.57E-02	190	1216	9.17E-03	310	3062	1.99E-03

71	367	7.75E-02	191	1366	4.72E-03	311	3062	1.48E-03
72	367	7.94E-02	192	1366	5.28E-03	312	3062	1.88E-03
73	576	2.75E-02	193	1367	3.94E-03	313	3062	3.09E-03
74	576	2.71E-02	194	1367	4.34E-03	314	3063	2.12E-03
75	576	1.92E-02	195	1367	4.01E-03	315	3065	1.74E-03
76	589	8.30E-02	196	1368	4.64E-03	316	3066	1.83E-03
77	589	8.28E-02	197	1369	9.94E-03	317	3066	2.55E-03
78	592	1.45E-02	198	1369	9.74E-03	318	3075	1.64E-03
79	633	5.29E-02	199	1372	8.17E-04	319	3076	1.62E-03
80	633	1.00E-01	200	1374	5.59E-03	320	3076	1.58E-03

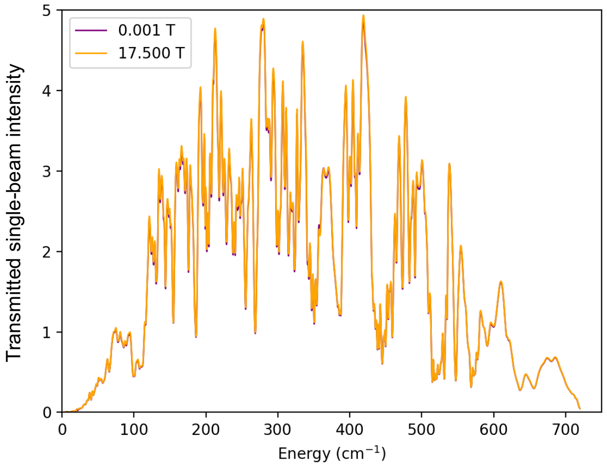

Figure S15. Far-IR transmission spectra at 0 and 17.5 T of a powder sample of **1** in the 0-720 cm^{-1} energy range.

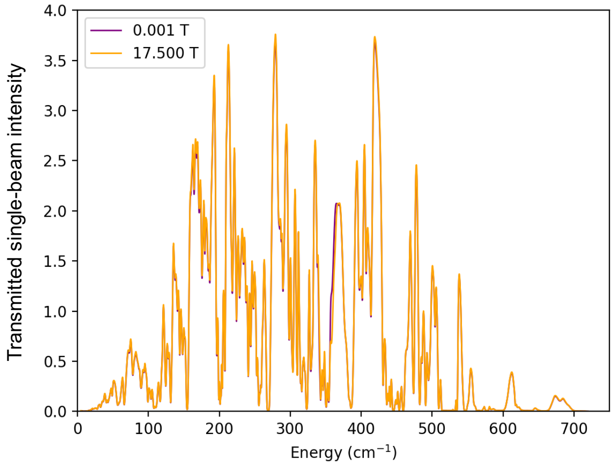

Figure S16. Far-IR transmission spectra at 0 and 17.5 T of **1-*B1*** in the 0-720 cm^{-1} energy range.

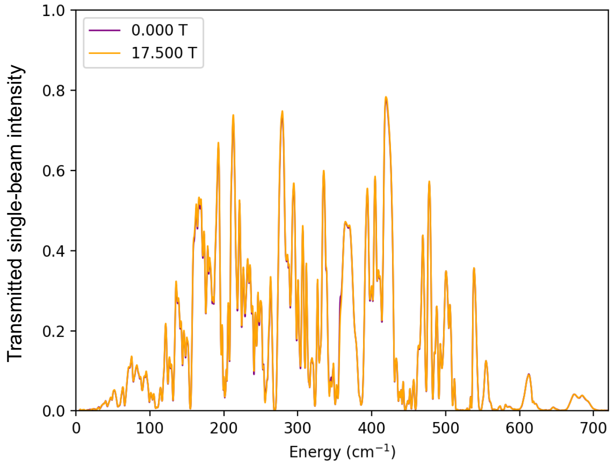

Figure S17. Far-IR transmission spectra at 0 and 17.5 T of **1-*B2*** in the 0-720 cm^{-1} energy range.

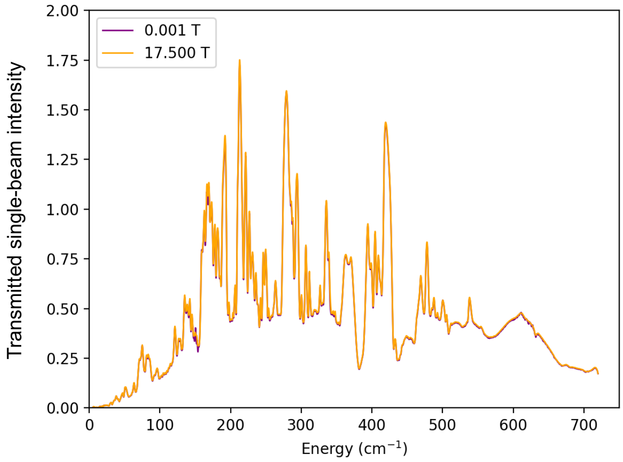

Figure S18. Far-IR transmission spectra at 0 and 17.5 T of **1-*B3*** in the 0-720 cm^{-1} energy range.

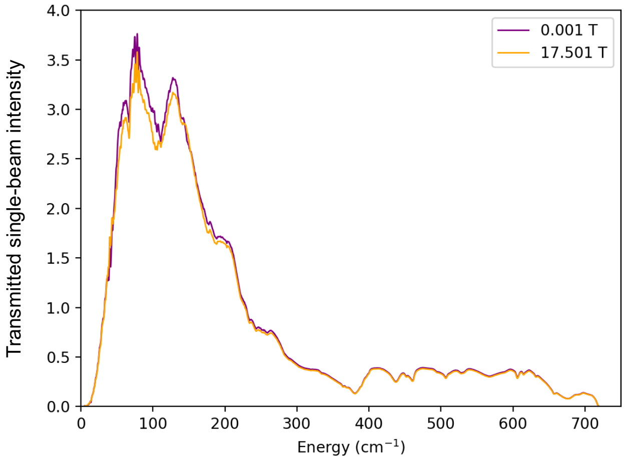

Figure S19. Far-IR transmission spectra at 0 and 17.5 T of **2** in the 0-720 cm^{-1} energy range.

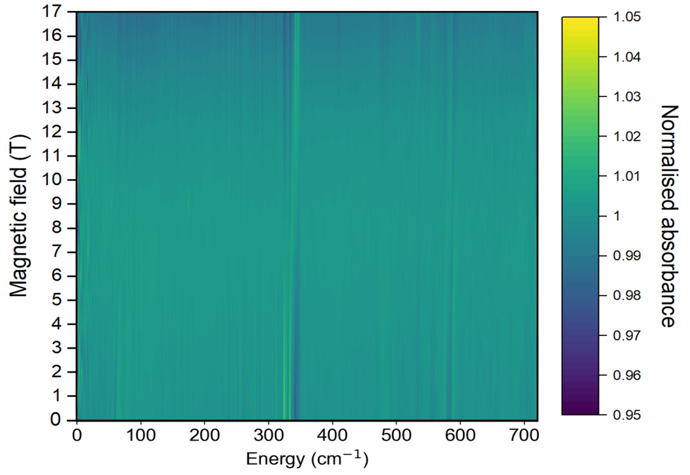

Figure S20. Experimental FIRMS heatmap of a powder sample of **1** in the 0-720 cm^{-1} energy range.

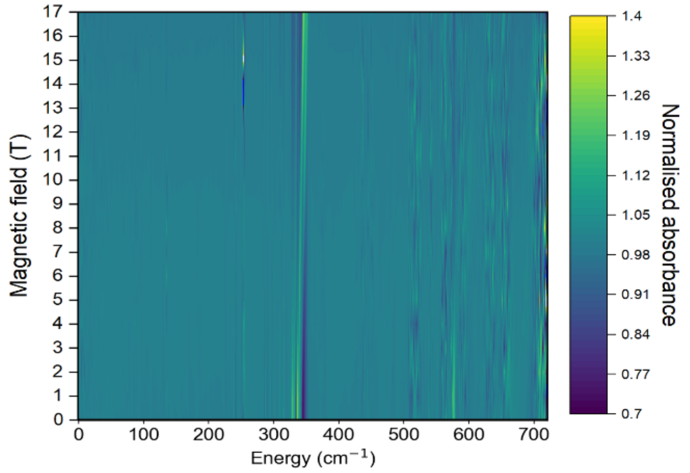

Figure S21. Experimental FIRMS heatmap of **1-*B1*** in the 0-720 cm^{-1} energy range.

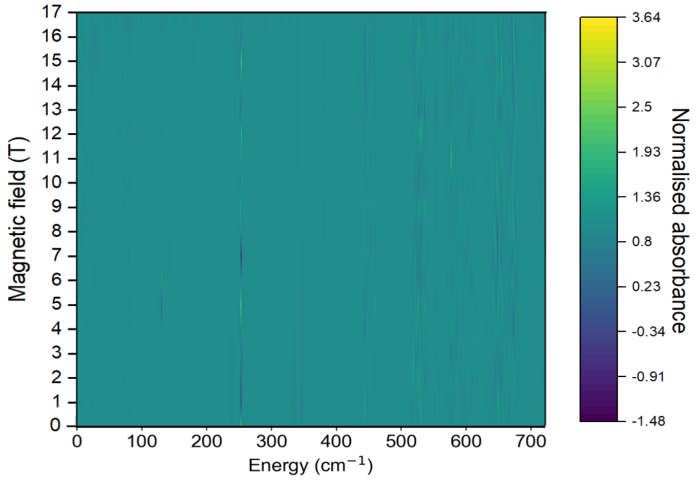

Figure S22. Experimental FIRMS heatmap of **1-*B2*** in the 0-720 cm^{-1} energy range.

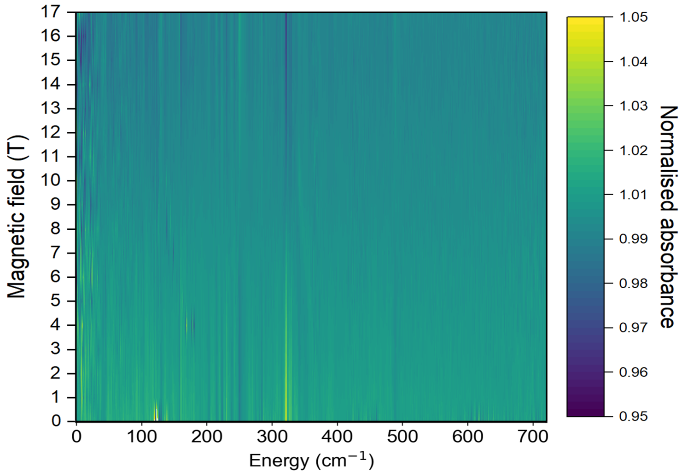

Figure S23. Experimental FIRMS heatmap of **1-*B3*** in the 0-720 cm^{-1} energy range.

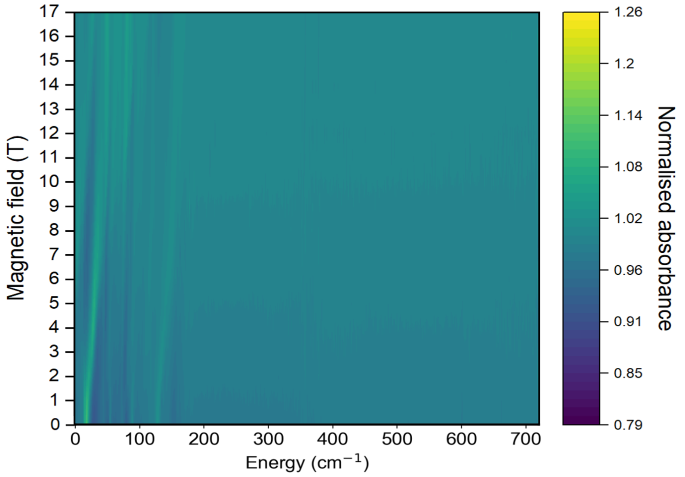

Figure S24. Experimental FIRMS heatmap of a powder sample of **2** in the 0-720 cm^{-1} energy range.

References

[1] I. F. Díaz-Ortega, J. Manuel Herrera, S. Dey, H. Nojiri, G. Rajaraman, E. Colacio, "The effect of the electronic structure and flexibility of the counteranions on magnetization relaxation in [Dy(L) 2 (H 2 O) 5] 3+ (L = phosphine oxide derivative) pentagonal bipyramidal SIMs" *Inorg. Chem. Front.* **2020**, *7*, 689–699.

[2] W. A. Herrmann, R. Anwander, F. C. Munck, W. Scherer, V. Dufaud, N. W. Huber, G. R. J. Artus, "Lanthanoiden-Komplexe, IX [1]. Reaktivitätsbestimmender Einfluß der Ligandenkonstitution bei Seltenerdamiden: Herstellung und Struktur sterisch überladener Alkoxid-Komplexe / Lanthanoid Complexes, IX [1]. Reactivity Control of Lanthanoid Amides through Ligand Effects: Synthesis and Structures of Sterically Congested Alkoxy Complexes" *Z. Für Naturforschung B* **1994**, *49*, 1789–1797.

[3] M. J. Frisch, G. W. Trucks, H. B. Schlegel, G. E. Scuseria, M. A. Robb, J. R. Cheeseman, G. Scalmani, V. Barone, B. Mennucci, G. A. Petersson, H. Nakatsuji, M. Caricato, X. Li, H. P. Hratchian, A. F. Izmaylov, J. Bloino, G. Zheng, J. L. Sonnenberg, M. Hada, M. Ehara, K. Toyota, R. Fukuda, J.

Hasegawa, M. Ishida, T. Nakajima, Y. Honda, O. Kitao, H. Nakai, T. Vreven, J. A. Montgomery Jr., J. E. Peralta, F. Ogliaro, M. Bearpark, J. J. Heyd, E. Brothers, K. N. Kudin, V. N. Staroverov, T. Keith, R. Kobayashi, J. Normand, K. Raghavachari, A. Rendell, J. C. Burant, S. S. Iyengar, J. Tomasi, M. Cossi, N. Rega, J. M. Millam, M. Klene, J. E. Knox, J. B. Cross, V. Bakken, C. Adamo, J. Jaramillo, R. Gomperts, R. E. Stratmann, O. Yazyev, A. J. Austin, R. Cammi, C. Pomelli, J. W. Ochterski, R. L. Martin, K. Morokuma, V. G. Zakrzewski, G. A. Voth, P. Salvador, J. J. Dannenberg, S. Dapprich, A. D. Daniels, Ö. Farkas, J. B. Foresman, J. V. Ortiz, J. Cioslowski, D. J. Fox **n.d.**

[4] E. Sigfridsson, U. Ryde, “Comparison of methods for deriving atomic charges from the electrostatic potential and moments” *J. Comput. Chem.* **1998**, *19*, 377–395.

[5] J. P. Perdew, K. Burke, M. Ernzerhof, “Generalized Gradient Approximation Made Simple” *Phys. Rev. Lett.* **1996**, *77*, 3865–3868.

[6] S. Grimme, “Density functional theory with London dispersion corrections” *Wiley Interdiscip. Rev. Comput. Mol. Sci.* **2011**, *1*, 211–228.

[7] T. H. Dunning, “Gaussian basis sets for use in correlated molecular calculations. I. The atoms boron through neon and hydrogen” *J. Chem. Phys.* **1989**, *90*, 1007–1023.

[8] G. Kresse, J. Hafner, “Ab initio molecular dynamics for liquid metals” *Phys. Rev. B* **1993**, *47*, 558–561.

[9] G. Kresse, J. Hafner, “Ab initio molecular-dynamics simulation of the liquid-metal--amorphous-semiconductor transition in germanium” *Phys. Rev. B* **1994**, *49*, 14251–14269.

[10] G. Kresse, J. Furthmüller, “Efficient iterative schemes for ab initio total-energy calculations using a plane-wave basis set” *Phys. Rev. B* **1996**, *54*, 11169–11186.

[11] G. Kresse, J. Furthmüller, “Efficiency of ab-initio total energy calculations for metals and semiconductors using a plane-wave basis set” *Comput. Mater. Sci.* **1996**, *6*, 15–50.

[12] P. E. Blöchl, “Projector augmented-wave method” *Phys. Rev. B* **1994**, *50*, 17953–17979.

[13] G. Kresse, D. Joubert, “From ultrasoft pseudopotentials to the projector augmented-wave method” *Phys. Rev. B* **1999**, *59*, 1758–1775.

[14] A. Togo, I. Tanaka, “First principles phonon calculations in materials science” *Scr. Mater.* **2015**, *108*, 1–5.

[15] A. Togo, “First-principles Phonon Calculations with Phonopy and Phono3py” *J. Phys. Soc. Jpn.* **2023**, *92*, 012001.

[16] I. Fdez. Galván, M. Vacher, A. Alavi, C. Angeli, F. Aquilante, J. Autschbach, J. J. Bao, S. I. Bokarev, N. A. Bogdanov, R. K. Carlson, L. F. Chibotaru, J. Creutzberg, N. Dattani, M. G. Delcey, S. S. Dong, A. Dreuw, L. Freitag, L. M. Frutos, L. Gagliardi, F. Gendron, A. Giussani, L. González, G. Grell, M. Guo, C. E. Hoyer, M. Johansson, S. Keller, S. Knecht, G. Kovačević, E. Källman, G. Li Manni, M. Lundberg, Y. Ma, S. Mai, J. P. Malhado, P. Å. Malmqvist, P. Marquetand, S. A. Mewes, J. Norell, M. Olivucci, M. Oppel, Q. M. Phung, K. Pierloot, F. Plasser, M. Reiher, A. M. Sand, I. Schapiro, P. Sharma, C. J. Stein, L. K. Sørensen, D. G. Truhlar, M. Ugandi, L. Ungur, A. Valentini, S. Vancoillie, V. Veryazov, O. Weser, T. A. Wesołowski, P.-O. Widmark, S. Wouters, A. Zech, J. P. Zobel, R. Lindh, “OpenMolcas: From Source Code to Insight” *J. Chem. Theory Comput.* **2019**, *15*, 5925–5964.

[17] G. Li Manni, I. Fdez. Galván, A. Alavi, F. Aleotti, F. Aquilante, J. Autschbach, D. Avagliano, A. Baiardi, J. J. Bao, S. Battaglia, L. Birnoschi, A. Blanco-González, S. I. Bokarev, R. Broer, R. Cacciari, P. B. Calio, R. K. Carlson, R. Carvalho Couto, L. Cerdán, L. F. Chibotaru, N. F. Chilton, J. R. Church, I. Conti, S. Coriani, J. Cuéllar-Zuquin, R. E. Daoud, N. Dattani, P. Decleva, C. De Graaf, M. G. Delcey, L. De Vico, W. Dobrautz, S. S. Dong, R. Feng, N. Ferré, M. Filatov(Gulak), L. Gagliardi, M. Garavelli, L. González, Y. Guan, M. Guo, M. R. Hennefarth, M. R. Hermes, C. E. Hoyer, M. Huix-Rotllant, V. K. Jaiswal, A. Kaiser, D. S. Kaliakin, M. Khamesian, D. S. King, V. Kochetov, M. Krośnicki, A. A. Kumaar, E. D. Larsson, S. Lehtola, M.-B. Lepetit, H. Lischka, P. López Ríos, M. Lundberg, D. Ma, S. Mai, P. Marquetand, I. C. D. Merritt, F. Montorsi, M. Mörchen, A. Nenov, V. H. A. Nguyen, Y. Nishimoto, M. S. Oakley, M. Olivucci, M. Oppel, D. Padula, R. Pandharkar, Q. M. Phung, F. Plasser, G. Raggi, E. Rebolini, M. Reiher, I. Rivalta, D. Roca-Sanjuán, T. Romig, A. A. Safari, A. Sánchez-Mansilla, A. M. Sand, I. Schapiro, T. R. Scott, J. Segarra-Martí, F. Segatta, D.-C. Sergentu, P. Sharma, R. Shepard, Y. Shu, J. K. Staab, T. P. Straatsma, L. K. Sørensen, B. N. C. Tenorio, D. G. Truhlar, L. Ungur, M. Vacher, V. Veryazov, T. A. Voß, O. Weser, D. Wu, X. Yang, D. Yarkony, C. Zhou, J. P. Zobel, R. Lindh, "The OpenMolcas *Web* : A Community-Driven Approach to Advancing Computational Chemistry" *J. Chem. Theory Comput.* **2023**, *19*, 6933–6991.

[18] B. O. Roos, R. Lindh, P.-Å. Malmqvist, V. Veryazov, P.-O. Widmark, "New Relativistic ANO Basis Sets for Transition Metal Atoms" *J. Phys. Chem. A* **2005**, *109*, 6575–6579.

[19] F. Aquilante, L. Gagliardi, T. B. Pedersen, R. Lindh, "Atomic Cholesky decompositions: A route to unbiased auxiliary basis sets for density fitting approximation with tunable accuracy and efficiency" *J. Chem. Phys.* **2009**, *130*, 154107.

[20] L. Ungur, L. F. Chibotaru, "Ab Initio Crystal Field for Lanthanides" *Chem. – Eur. J.* **2017**, *23*, 3708–3718.

[21] J. K. Staab, N. F. Chilton, "Analytic Linear Vibronic Coupling Method for First-Principles Spin-Dynamics Calculations in Single-Molecule Magnets" *J. Chem. Theory Comput.* **2022**, *18*, 6588–6599.

[22] J. G. C. Kragskow, J. Marbey, C. D. Buch, J. Nehrkorn, M. Ozerov, S. Piligkos, S. Hill, N. F. Chilton, "Analysis of vibronic coupling in a 4f molecular magnet with FIRMS" *Nat. Commun.* **2022**, *13*, 825.

[23] J. Nehrkorn, "General Magnetic Transition Dipole Moments for Electron Paramagnetic Resonance" *Phys. Rev. Lett.* **2015**, *114*, DOI 10.1103/PhysRevLett.114.010801.

[24] D. Reta, J. G. C. Kragskow, N. F. Chilton, "Ab Initio Prediction of High-Temperature Magnetic Relaxation Rates in Single-Molecule Magnets" *J. Am. Chem. Soc.* **2021**, *143*, 5943–5950.

[25] J. Mulak, M. Mulak, "The maximal axial components in the equivalent parametrizations of crystal-field Hamiltonians" *Phys. Status Solidi B* **2006**, *243*, 2796–2810.
